# The role of angiopoietin-like protein 4 in phenylephrine-induced cardiomyocyte hypertrophy

**DOI:** 10.1042/BSR20171358

**Published:** 2019-07-29

**Authors:** Yu Sun, Yi Li, Chen Liu, Ruicong Xue, Bin Dong, Huiling Huang, Longyun Peng, Jun Liu, Yugang Dong

**Affiliations:** 1 Department of Cardiology, the First Affiliated Hospital of Sun Yat-Sen University, Guangzhou, Guangdong, China, 510080; 2 Key Laboratory on Assisted Circulation, Ministry of Health, Guangzhou, Guangdong, China, 510080

**Keywords:** ANGPTL4, cardiomyocyte hypertrophy, CPT-1, PPARα, JNK1/2

## Abstract

Angiopoietin-like protein 4 (ANGPTL4) is a multifunctional secreted protein that can be induced by fasting, hypoxia and glucocorticoids. ANGPTL4 has been associated with a variety of diseases; however, the role of ANGPTL4 in cardiac hypertrophy remains poorly understood. In our study, we aimed to explore the effect of ANGPTL4 on phenylephrine-induced cardiomyocyte hypertrophy. Our results showed that knockdown of ANGPTL4 expression significantly exacerbated cardiomyocyte hypertrophy, as demonstrated by increased hypertrophic marker expression, including ANP and cell surface area. Moreover, significantly reduced fatty acid oxidation, as featured by decreased CPT-1 levels, was observed in hypertrophic cardiomyocytes following ANGPTL4 down-regulation. Furthermore, knockdown of ANGPLT4 led to down-regulated expression of peroxisome proliferator-activated receptor α (PPARα), which is the key regulator of cardiac fatty acid oxidation. In addition, ANGPTL4 silencing promoted the activation of JNK1/2, and JNK1/2 signaling blockade could restore the level of PPARα and significantly ameliorate the ANGPTL4 knockdown-induced cardiomyocyte hypertrophy. Therefore, our study demonstrated that ANGPTL4 regulates PPARα through JNK1/2 signaling and is required for the inhibition of cardiomyocyte hypertrophy.

## Introduction

Cardiac hypertrophy is a compensatory mechanism in response to cardiac overload or injury. Nevertheless, sustained cardiac hypertrophy is regarded as a leading cause of heart failure [1]. Under normal circumstances, the heart is capable of utilizing energy substrates to satisfy its high energy demand. It is well established that fatty acids are the predominant energy substrates used in the adult myocardium and produce more energy than other metabolic substrates [2]. However, in response to pathological hypertrophy, the myocardium relies more on glucose metabolism than on fatty acid oxidation (FAO), leading to a decreased supply of energy [3]. Over the last decade, many studies have suggested that decreased myocardial FAO is likely a key pathological mechanism that contributes to the transition to heart failure [4–6]. Therefore, therapeutic strategies that prevent the down-regulation of FAO in the hypertrophied heart are highly desirable, but the mechanism is poorly understood.

ANGPTL4 (angiopoietin-like 4, also known as HFARP, FIAF and PGAR) is a multifunctional secreted protein that belongs to a family consisting of seven members (ANGPTL1–7). ANGPTL4, which comprises an N-terminal coiled-coil domain and a C-terminal fibrinogen-like domain, has been implicated in numerous diseases, including cardiovascular disease, obesity, diabetes, nephrotic syndrome, cancer metastasis, wound repair, inflammation and arthritis [7]. Many studies have shown that ANGPTL4 is a key regulator of metabolism and could modulate lipid metabolism by inhibiting the activity of lipoprotein lipase (LPL) [8], an enzyme responsible for the hydrolysis of triglycerides (TG) contained in lipoproteins. However, whether ANGPTL4 can regulate cardiac hypertrophy remains poorly understood.

Thus, in our present study, we aimed to discover the effects of ANGPTL4 on cardiac hypertrophy and explore the underlying mechanism. Our data suggest that ANGPTL4 is required for the inhibition of cardiomyocyte hypertrophy, possibly through the regulation of the expressions of FAO-related genes.

## Materials and methods

### Reagents

Monoclonal antibodies against ERK1/2, phospho-ERK1/2, JNK1/2, phospho-JNK1/2, p38 and phospho-p38 were purchased from Cell Signaling Technology (Massachusetts, U.S.A.). Anti-ANGPTL4 antibody was obtained from Sigma (St. Louis, MO, U.S.A.). DMEM/F12 and fetal bovine serum (FBS) were purchased from HyClone. Collagenase and trypsin were purchased from Gibco (Grand Island, NY, U.S.A.). Cell lysis buffer was obtained from Cell Signaling Technology (Massachusetts, U.S.A.). Recombinant human ANGPTL4 was from Abnova. Phenylephrine (PE) was purchased from Tokyo Chemical Industry. TRIzol and JNK inhibitor (SP600125) was obtained from Sigma (St. Louis, MO, U.S.A.).

### Animals

All of the experimental protocols complied with the guide for the care and use of laboratory animals published by the Ethics Committee on Clinical Research and Animal Research of the First Affiliated Hospital of Sun Yat-Sen University. Eight- to ten-week-old male C57BL/6J mice weighing 24–26 g were used in the current research study. Cardiac hypertrophy was induced by pressure overload, which was achieved by descending aortic banding (AB) as previously described [9]. Following anesthesia by intraperitoneal injection of 1.5% pentobarbital, the left thorax of C57BL/6J mice was opened at the second intercostal space, and a 7–0 silk suture ligature was tied around the descending aorta against a 26-gauge needle. Then, the needle was quickly removed. A similar surgery was performed on the sham-operated mice with the exception of AB.

### Neonatal rat ventricular cardiomyocyte cultures and siRNA and recombinant ANGPTL4 transfection

Primary cultures of cardiomyocytes were obtained from the Experimental Animal Facility of Sun Yat-Sen University and were prepared from 1- to 2-day-old Sprague-Dawley rats as previously described [10]. siRNA transfection was performed using Lipofectamine™ RNAiMAX (Invitrogen, Carlsbad, CA) according to the manufacturer’s instructions. The sequence of the siRNA targeting ANGPTL4 (si-ANGPTL4) was as follows: 5′GCAGCCAUUCCAAUCUAAAdTdT3′. Thirty-six hours after being seeded, cardiomyocytes were transfected with scrambled siRNA (si-scramble) (50 nmol/l) or ANGPTL4-specific siRNA (50 nmol/l) in serum-free medium for 12 h. After another 12 h of serum-free medium starvation, the cardiomyocytes were treated with PE (50 μM) to induce cardiomyocyte hypertrophy. Thirty-six hours after being seeded, recombinant ANGPTL4 (0.5 μg/ml) was added into cardiomyocytes in serum-free medium for 24 h. Then, the cardiomyocytes were treated with PE (50 μM) to induce cardiomyocyte hypertrophy.

### Administration of JNK inhibitor

JNK inhibitor (SP600125) was dissolved in dimethylsulfoxide (DMSO) to a final concentration of 10 μM. The cardiomyocytes were plated at a density of 1 × 10^6^ cells/well in six-well plates. Following 36 h of incubation, JNK inhibitor (10 nM) or DMSO was added to each well 1 h prior to treatment with siRNA.

### RNA isolation and quantitative real-time PCR (q-PCR)

RNA isolation and q-PCR were performed as previously described [11]. The primers used are listed in Table 1. Q-PCR was performed under the following conditions: 95°C for 5 min followed by 39 cycles of 95°C for 10 s, 60°C for 20 s, and 72°C for 20 s, using a LightCycler 480 thermal cycler (Roche Diagnostics GmbH, Manheim, Germany). Data were normalized using GAPDH as an internal control.

**Table 1:** Sequences of oligonucleotide primers (forward and reverse) used for PCR

Target	Forward (5′-3′orientation)	Reverse (5′-3′orientation)
ANGPTL4	AGCTCAAGGCTCAAAACAGCA	CTTTCCCCTCGAAGTCTTGTCT
CPT-1	TGCAGTCGACTCACCTTTCC	TCAAAGAGCTCCACCTGCTG
CD36/FAT	TACTCTCTCCTCGGATGGCT	AGCACTTGCTTCTTGCCAAC
PPARα-rat	GTCCTCTGGTTGTCCCCTTG	GTCAGTTCACAGGGAAGGCA
ANP-rat	TGAGCCGAGACAGCAAACATC	AGGCCAGGAAGAGGAAGAAGC
GAPDH-rat	ACAGCAACAGGGTGGTGGAC	TTTGAGGGTGCAGCGAACTT
PPARα-mouse	TGCCTTCCCTGTGAACTGAC	TGGGGAGAGAGGACAGATGG
ANP-mouse	GCTTCCTGCCTTCATCTATCAC	TGAAAAGGGTGAGGATCTACCT
GAPDH- mouse	GTTGTCTCCTGCGACTTCAAC	GCTGTAGCCGTATTCATTGTCA

### Western blotting analysis

Western blotting was performed according to our previous study [12]. Quantitative analysis was performed using the Quantity One software. The membrane was blocked with blocking buffer (1× TBS, 0.1% Tween-20, 5% BSA) for 1 h at room temperature and then incubated overnight at 4°C with anti-phosphorylated-ERK1/2 (1:3000 dilution), anti-total-ERK1/2 (1:3000 dilution), anti-phosphorylated-JNK1/2 (1:1000 dilution), anti-total-JNK1/2 (1:1000 dilution), anti-phosphorylated-p38 (1:1000 dilution), anti-total-p38 (1:1000 dilution), anti-ANGPTL4 (1:1000 dilution) or anti-GAPDH (1:10000 dilution) primary antibodies. Then, the membrane was washed with TBS-T and incubated with secondary antibodies (1:10000 dilution, Protein-tech Group, Wuhan, China) at 37°C for 1 h. The immune complex was detected with an enhanced chemiluminescence system (Millipore, Massachusetts, U.S.A.) and exposed to X-ray film.

### Measurement of the surface area of the cardiomyocytes

The cardiomyocytes were exposed to PE for 24 h and then fixed using 4% paraformaldehyde. Subsequently, 50–100 cardiomyocytes in each group were randomly selected, and the cell surface area was analyzed using the Image-Pro Plus software.

### Immunofluorescence staining

Immunofluorescence staining was performed as previously described [12]. Mouse polyclonal anti-troponin I (1:50, Santa Cruz) was used as the primary antibody, and the immune complexes were detected using Cy3-conjugated secondary antibodies (1:100, Protein-tech Group). The nuclei were stained with DAPI (0.5 mg/ml, Sigma). The images were obtained at 600× using a Nikon A1+ confocal microscope.

### Statistical analysis

All of the data were expressed as the mean ± standard error of the mean (SEM) from at least three independent experiments. The differences between the means were evaluated using one-way or two-way ANOVA. Statistical significance was established at *P*<0.05. All of the statistical analyses were performed using SPSS13.0 software.

## Results

### ANGPTL4 expression is increased in PE-induced hypertrophic cardiomyocytes

**Figure 1:**
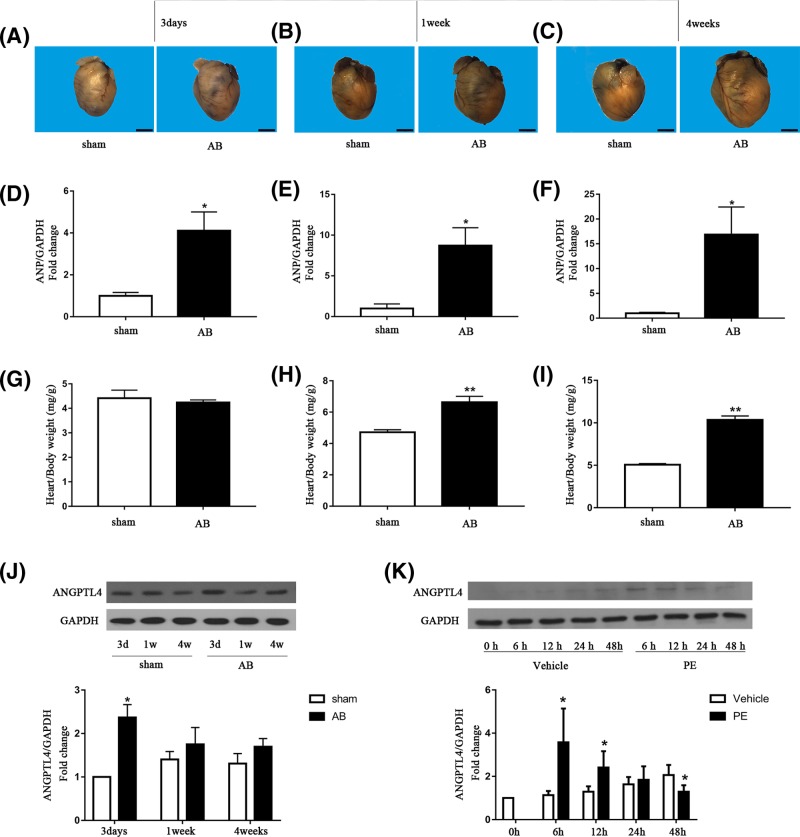
Effect of AB or PE on ANGPTL4 expression in cardiomyocytes (**A–C**) Representative images of the mouse heart at 3 days, 1 week and 4 weeks after AB or sham surgery. (**D–F**) The effect of AB on ANP mRNA expression was determined by q-PCR, and GAPDH was used as an internal control. (**G–I**) Quantitative analysis of the heart/body weight of mice at 3 days, 1 week and 4 weeks after aortic banding or sham surgery. (**J**) Western blots showing ANGPTL4 and GAPDH expression in mouse hearts at the indicated time after sham or AB surgery, and quantitative analysis of the above blots. (**K**) Western blots showing ANGPTL4 and GAPDH expression in cultured cardiomyocytes after vehicle or PE incubation for the indicated time, and quantitative analysis of the above blots. GAPDH was used as an internal control. *P<0.05 versus the corresponding control group. **P<0.01 versus the corresponding control group. The results represent three to five independent experiments; *n* = 3–5.

To explore whether ANGPTL4 plays a role in pressure overload- or PE-induced cardiomyocyte hypertrophy, we detected ANGPTL4 protein expression in cardiomyocytes 3 days, 1 week and 4 weeks after AB or sham surgery *in vivo* and after 6, 12 and 24 h of PE incubation *in vitro*. As shown in Figure 1A–I, gross heart size, ANP mRNA expression and the heart/body weight increased with time after AB surgery, whereas no significant change in heart size, ANP mRNA expression and heart/body weight was observed in the mice that underwent sham surgery. Compared with the sham group, the level of ANGPTL4 was increased 3 days after AB surgery and gradually declined thereafter (Figure 1G). Similarly, *in vitro* experiments showed that the level of ANGPTL4 was increased at 6 and 12 h after PE treatment and then gradually declined over the next 12 h (Figure 1H). Therefore, our data suggest that changes in ANGPTL4 expression might play a role in the process of AB- or PE-induced cardiac hypertrophy.

### ANGPTL4 protects against the PE-induced hypertrophic response in cardiomyocytes

**Figure 2:**
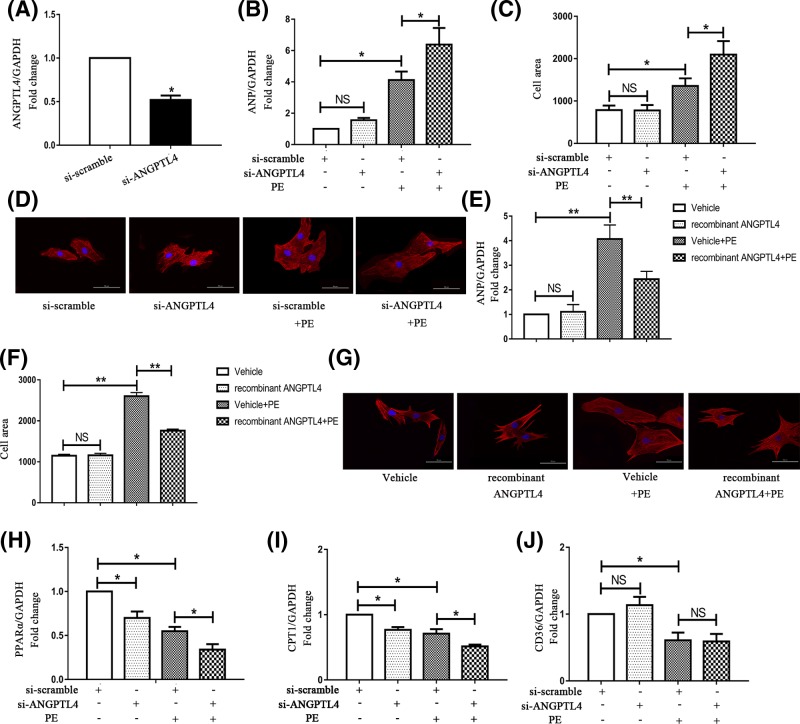
Effects of ANGPTL4 knockdown and recombinant ANGPTL4 treatment on PE-induced cardiomyocyte hypertrophy and the genes (PPARα, CPT-1 and CD36) involved in the regulation of FAO Cardiomyocytes were transfected with siRNA and incubated with or without PE for 24 h in serum-free medium. (**A**) The siRNA-mediated knockdown of ANGPTL4 was confirmed by q-PCR. (**B**) The effect of siRNA-mediated knockdown of ANGPTL4 on ANP mRNA expression was determined by q-PCR, and GAPDH was used as an internal control. (**C**) The effect of silencing ANGPTL4 on the cell surface area. After siRNA transfection, cardiomyocytes were treated with or without PE for 24 h. (**D** and **G**) Cardiomyocytes were stained with troponin I, and the nuclei were stained with DAPI. (**E**) The effect of recombinant ANGPTL4 on ANP mRNA expression was detected by q-PCR. (**F**) The effect of recombinant ANGPTL4 on the cell surface area. After siRNA transfection, the expression levels of PPARα (**H**), CPT-1 (**I**) and CD36 (**J**) were detected by q-PCR, and GAPDH was used as an internal control. *P<0.05 versus the corresponding control group; NS indicates no significance versus the corresponding control group. Each of the experiments was repeated four to seven times; *n* = 4–7.

To determine whether ANGPTL4 regulates the development of cardiomyocyte hypertrophy, we first knocked down ANGPTL4 expression in cardiomyocytes using siRNA transfection. The silencing effect was examined by q-PCR, and the expression level of ANGPTL4 was decreased to approximately 50% after siRNA transfection (Figure 2A). Furthermore, hallmark parameters of cardiac hypertrophy, including ANP mRNA expression and cardiomyocyte surface area, were detected. As shown in Figure 2B,D, knockdown of ANGPTL4 did not affect the basal levels of ANP or the cell surface area. Under PE stress, cardiomyocytes transfected with ANGPTL4 siRNA (si-ANGPTL4) exhibited a more significant hypertrophic phenotype than those transfected with control siRNA (si-scramble). Moreover, the mRNA expression level of ANP and the cell surface area were increased by approximately 50% in the si-ANGPTL4 group compared with the control group. These data suggested that knockdown of ANGPTL4 expression exacerbated PE-induced cardiomyocyte hypertrophy.

To further evaluate the inhibitory effect of ANGPTL4 on cardiac hypertrophy, we examined the effect of exogenous ANGPTL4 on cardiac hypertrophy. We next investigated the levels of ANP and the cell surface area of cardiomyocytes treated with recombinant human ANGPTL4 compared with those of cardiomyocytes treated with vehicle under hypertrophic stress. Treatment with recombinant human ANGPTL4 abolished the increase in ANP levels (41%) (Figure 2E) compared with treatment with vehicle. Similarly, the cell surface area of the cardiomyocytes treated with recombinant ANGPTL4 was markedly decreased compared with that of controls (32.3%) (Figure 2F,G). Therefore, these results verified that ANGPTL4 is a potent inhibitor of PE-induced cardiac hypertrophy.

### Down-regulation of ANGPTL4 impairs the expressions of fatty acid metabolism related genes in hypertrophic cardiomyocytes

As FAO is one of the pivotal mechanisms involved in the development of cardiac hypertrophy, we explored whether ANGPTL4 affected fatty acid metabolism in hypertrophic cardiomyocytes. Peroxisome proliferator-activated receptor α (PPARα, the major regulator of FAO) and its downstream effectors, CPT-1 and FAT/CD36 [13,14], were detected. CPT1 is the rate-limiting step of FAO and imports long-chain fatty acids (FAs) across the mitochondrial membrane; FAT/CD36 is regarded as an FA transporter. As shown in Figure 2H–J, we confirmed that PE-induced cardiomyocyte hypertrophy led to a notable decrease in PPARα, CPT-1 and FAT/CD36 expression, suggesting that impaired FAO is involved in the process of cardiomyocyte hypertrophy. Compared with the cardiomyocytes transfected with control siRNA, the cardiomyocytes transfected with si-ANGPTL4 demonstrated a significant decrease in the expression of PPARα and CPT-1 both at baseline and under PE stress. This finding suggested that ANGPTL4 may be involved in the regulation of the expressions of FAO-related genes in cardiomyocytes with or without PE treatment. However, no differential expression of FAT/CD36 was observed following knockdown of ANGPTL4. Overall, these data demonstrated that down-regulation of ANGPTL4 decreased the expression of PPARα and CPT-1 in hypertrophic cardiomyocytes, indicating that reduced expressions of FAO-related genes might contribute to the exacerbated hypertrophy caused by ANGPTL4 knockdown.

### The roles of the MAPK signaling pathway in the ANGPTL4-mediated amelioration of cardiomyocyte hypertrophy

**Figure 3:**
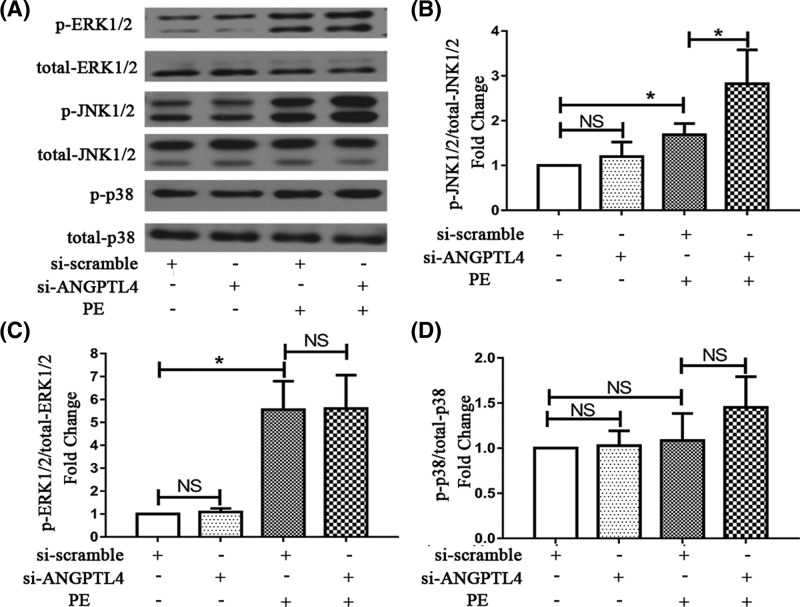
Effect of ANGPTL4 knockdown on MAPK signaling (**A**) The MAPK bands were detected using Western blot analysis after PE incubation for 30 min. Quantitative analysis of phosphorylated and total ERK1/2 (**B**), JNK1/2 (**C**) and p38 (**D**). *P<0.05 versus the corresponding control group. NS indicates no significance versus the corresponding control group. Each of the experiments was repeated six times; *n*=6.

In previous studies, inhibition of MAPK signaling was demonstrated to reduce PPARα activity [15]. Simultaneously, our previous study showed that PPARα could ameliorate cardiac hypertrophy and that PPARα expression could be induced by the activation of ERK1/2 signaling pathways [16]. Additionally, induction of ANGPTL4 expression was shown by Stapleton to significantly activate the ERK1/2 and JNK1/2 signaling pathways [17]. Therefore, to explore the roles of the MAPK signaling pathway in the ANGPTL4-mediated amelioration of cardiomyocyte hypertrophy, we further examined whether knockdown of ANGPTL4 promoted hypertrophy by decreasing PPARα through the MAPK signaling pathways. As shown in Figure 3A–D, PE treatment significantly induced the phosphorylation of ERK1/2 and JNK1/2. Transfection with si-ANGPTL4 did not affect the activation of MAPKs without PE treatment but further increased the phosphorylation of JNK1/2 under PE stress. However, ERK1/2 and p38 activation was not affected by ANGPTL4 knockdown. Therefore, JNK1/2 might be involved in the ANGPTL4-mediated regulation of cardiomyocyte hypertrophy.

**Figure 4:**
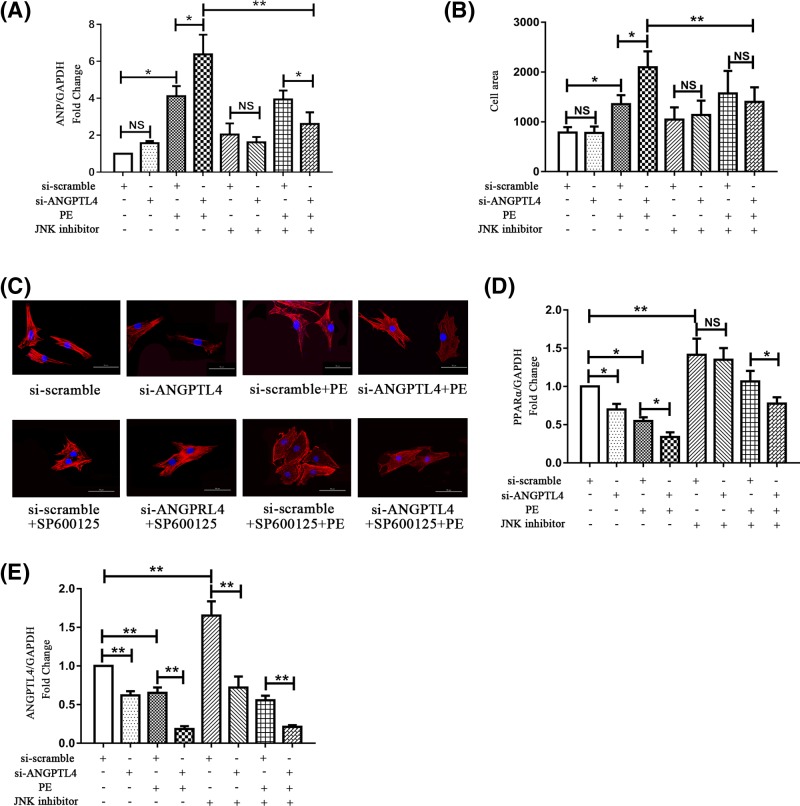
Effect of JNK inhibitor on ANGPTL4-mediated cardiomyocyte hypertrophy JNK inhibitor (SP600125) was added to cardiomyocytes 1 h prior to treatment with siRNA followed by treatment with or without PE for 24 h. (**A**) The effect of JNK inhibitor and siRNA on ANP mRNA expression was determined by q-PCR, and GAPDH was used as an internal control. (**B**) The effect of JNK inhibitor and ANGPTL4 knockdown on the cell surface area. (**C**) Cardiomyocytes were stained with troponin I, and the nuclei were stained with DAPI. (**D**) The expression levels of PPARα were detected by q-PCR; GAPDH was used as an internal control. (**E**) The effect of JNK inhibitor and ANGPTL4 knockdown on ANGPTL4 mRNA expression was detected by q-PCR. *P<0.05 versus the corresponding control group. **P<0.01 versus the corresponding control group. NS indicates no significance versus the corresponding control group. Each of the experiments was repeated three to six times; *n*=3–6.

**Figure 5:**
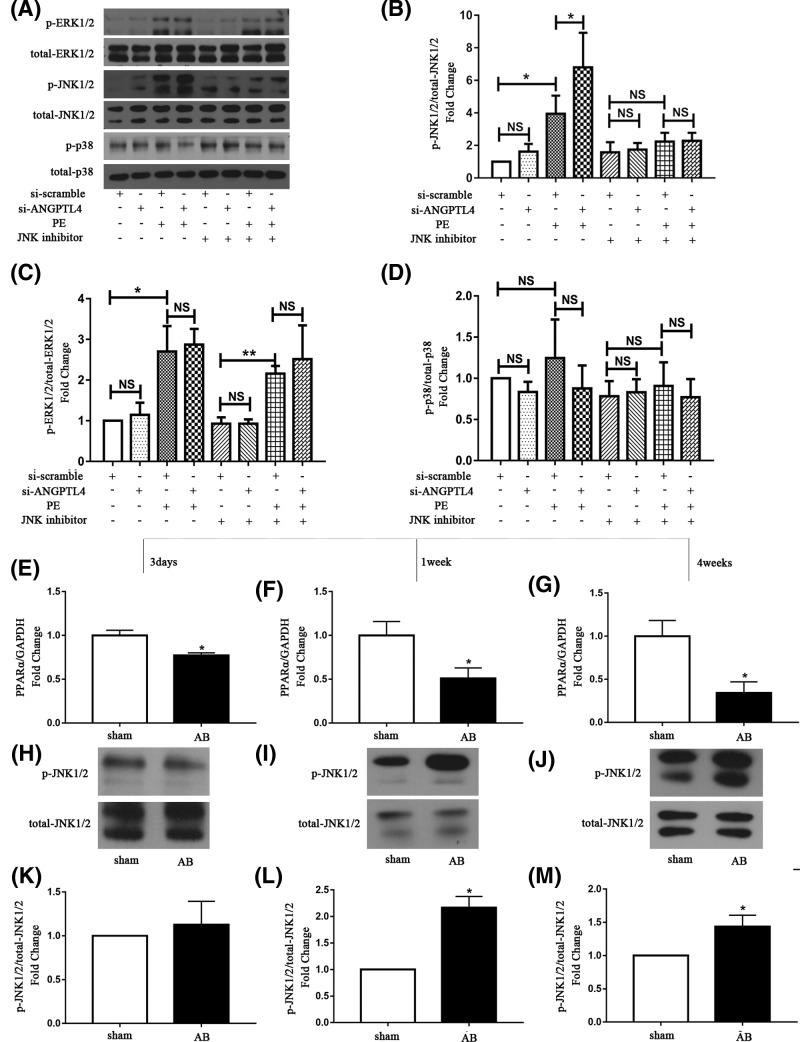
Effect of JNK inhibitor on MAPK signaling following ANGPTL4 knockdown JNK inhibitor (SP600125) was added to cardiomyocytes 1 h prior to treatment with siRNA followed by treatment with or without PE for 24 h. (**A**) The MAPK bands were detected using Western blot analysis after PE incubation for 30 min. Quantitative analysis of phosphorylated and total JNK1/2 (**B**), ERK1/2 (**C**) and p38 (**D**). (**E–G**) The effect of AB on PPARα mRNA expression was determined by q-PCR, and GAPDH was used as an internal control. (**H–J**) The JNK1/2 bands were detected using Western blot analysis after AB surgery. (**K–M**) Quantitative analysis of phosphorylated and total JNK1/2. *P<0.05 versus the corresponding control group. NS indicates no significance versus the corresponding control group. Each of the experiments was repeated three to seven times; *n*=3–7.

Previous studies have demonstrated that JNK1/2 signaling could inhibit PPARα signaling in the high-fat diet-induced fatty liver [18]. Thus, we investigated whether JNK1/2 played a role in the ANGPTL4-mediated regulation of cardiomyocyte hypertrophy. The JNK1/2 inhibitor SP600125 (10 nM) was used to block JNK1/2 before siRNA transfection. As shown in Figure 4A–C, treatment with JNK1/2 inhibitor significantly blocked the pro-hypertrophic effect of ANGPTL4 knockdown on PE-induced hypertrophic cardiomyocytes, decreasing ANP expression levels and cell surface area compared with the corresponding control group. Furthermore, as shown in Figure 4D, the expression level of PPARα was increased after JNK1/2 inhibitor treatment with or without si-ANGPTL4 transfection and PE stress, indicating that blockage of JNK1/2 markedly restored the level of PPARα. Additionally, the change in ANGPTL4 expression exhibited a similar trend as the change in PPARα mRNA expression (Figure 4E). Meanwhile, the effect of JNK inhibitor on the activation of MAPKs (ERK1/2, p38 and JNK1/2) was examined by Western blot. As shown in Figure 5A–D, activation of the JNK1/2 signaling pathway was significantly blocked by SP600125 treatment in the presence of PE stress, whereas JNK1/2 inhibitor had almost no influence on the ERK1/2 and p38 signaling pathways. Additionally, in *vivo* study showed the expression of PPARα decreased with time after AB surgery (Figure 5E–G). Pressure overload significantly induced the phosphorylation of JNK1/2 1 week after surgery and weakly induced 4 weeks after surgery. However, JNK1/2 was not activated 3 days after surgery (Figure 5H–M). The opposite changes of PPARα and JNK1/2 also suggest PPARα was regulated by JNK1/2 signaling in the process of AB-induced cardiac hypertrophy. Consequently, these data demonstrate that JNK1/2 signaling might mediate the pro-hypertrophic effects of ANGPTL4-knockdown on cardiomyocytes by regulating PPARα.

## Discussion

In our present study, we investigated the role of ANGPTL4 in PE-induced cardiomyocyte hypertrophy. We discovered that knockdown of ANGPTL4 aggravated the development of cardiomyocyte hypertrophy induced by PE. Meanwhile, decreased levels of PPARα and CPT-1 were observed in cardiomyocytes following ANGPTL4 knockdown in the absence or presence of PE, which suggested that down-regulation of ANGPTL4 might exacerbate cardiomyocyte hypertrophy through change the expressions of FAO-related genes. Furthermore, blocking JNK1/2 signaling ameliorated the exacerbated cardiomyocyte hypertrophy and the reduced PPARα expression level induced by down-regulation of ANGPTL4, indicating that JNK1/2 is a pivotal signaling molecule involved in the inhibition of cardiomyocyte hypertrophy by ANGPTL4.

It is well known that cardiac metabolism undergoes reprogramming in response to pathological hypertrophy, as characterized by decreased FAO and increased glucose utilization. Decreased dependence on FAs for energy production is a key pathological mechanism that contributes to the transition to heart failure [19]. Therefore, new targets that prevent the inhibition of FAO in the hypertrophied heart are highly desirable. ANGPTL4, a secretory protein, is predominantly expressed in the liver, adipose tissue and heart. The major function of ANGPTL4 is in lipid metabolism; ANGPTL4 is capable of inhibiting LPL activity by converting lipoprotein lipase to inactive monomers [20], which leads to a decrease in plasma TG level and reduced lipoprotein catabolism [21]. Further study showed that the N-terminal domain of ANGPTL4 was responsible for its inhibitory effect on LPL [22]. Therefore, based on the effect of ANGPTL4 on energy metabolism, we speculated that ANGPTL4 might participate in the regulation of cardiac hypertrophy.

To confirm our hypothesis, we first determined how ANGPTL4 expression is changed in cardiac hypertrophy. We discovered that the protein expression level of ANGPTL4 is increased at the onset of pressure overload- or PE-induced cardiac hypertrophy and declines over time. This finding suggested that changes in ANGPTL4 expression might be involved in the process of cardiac hypertrophy. To verify this speculation, specific siRNA was used to silence ANGPTL4. In hypertrophic cardiomyocytes, down-regulation of ANGPTL4 resulted in a further increase of hypertrophic markers (ANP expression and cell surface area). In contrast, the addition of exogenous recombinant ANGPTL4 abolished PE-induced hypertrophy. Taken together, we concluded that ANGPTL4 has a protective effect on the process of cardiomyocyte hypertrophy and that it may protect against hypertrophic stress.

It is well established that metabolism is altered in the hypertrophic myocyte. In general, FAO is normally the major energy source that sustains contractile function, whereas in response to pathological hypertrophy, the myocardium relies more on glucose metabolism, leading to a lower supply of energy [3]. Furthermore, PPARα, a member of the ligand-activated nuclear receptor superfamily, is a principal transcriptional regulator of FAO [23]. PPARα is widely expressed in tissues, such as the heart, that depend on FAO as a primary energy source [24]. PPARα is a critical regulator of myocardial metabolism, and increasing studies have suggested that abnormal regulation of PPARα is related to metabolic disturbances in the heart [25–27]. Moreover, decreased expression of PPARα has been reported to be associated with the development of pathological cardiac hypertrophy, and abnormalities in metabolism have been shown to be involved in that process [28,29]. Liang et al. [25] found that PPARα agonists could inhibit neonatal rat cardiomyocyte hypertrophy. Furthermore, Smeets et al. [28] discovered that knockout of PPARα could exacerbate chronic pressure overload-induced cardiac hypertrophy. Additionally, ANGPTL4 was shown by many independent groups to regulate PPARα [30–32]. These evidence indicate that ANGPTL4 may be effective in inhibiting cardiac hypertrophy via the regulation of PPARα. CPT-1, the downstream target of PPARα, is an essential enzyme in fatty acid metabolism [33] that converts long-chain acyl CoA to long-chain acylcarnitine. Long-chain acylcarnitine is subsequently shuttled into the mitochondria. Once in the mitochondrial matrix, long-chain acylcarnitine is converted back to long-chain acyl CoA, which subsequently participates in FAO. Thus, the regulation of CPT-1 expression by PPARα may influence the quantity of fatty acid that is shuttled into the mitochondria. As a rate-limiting enzyme of FAO, CPT-1 is positively correlated with energy production by FAO [33]. In our previous studies, we showed that PPARα could attenuate PE-induced cardiomyocyte hypertrophy by activating the ERK1/2 signaling pathways [16]. Consistent with our previous studies, we discovered that PPARα expression is decreased in cardiomyocytes transfected with si-ANGPTL4 under PE stress, which suggested that PPARα could be one of the targets of ANGPTL4 in the regulation of cardiac hypertrophy. Moreover, ANGPTL4 silencing reduced the basal CPT-1 level and further blocked its expression during cardiomyocyte hypertrophy, suggesting that ANGPTL4 could be an important regulator of FAO by negatively regulating PPARα/CPT-1 in cardiomyocytes.

Based on previous studies, MAPK signaling pathways are implicated in the regulation of PPARα. In our previous studies, we have demonstrated that activation of ERK1/2 induced PPARα [16]. In addition, activation of JNK1/2 leads to decreased expression of PPARα, which results in decreased the expressions of FAO-related genes [17]. To elucidate how ANGPTL4 regulates PPARα, we explored the effects of MAPKs in that process. In the present study, we discovered that treatment with PE stimulated the phosphorylation of ERK1/2 and JNK1/2. Additionally, activation of JNK1/2 was further increased in cardiomyocytes following down-regulation of ANGPTL4 under PE stress. However, activation of ERK1/2 and p38 was not affected by ANGPTL4 silencing, implying that ANGPTL4 mainly affects hypertrophy via JNK1/2 signaling. Therefore, a JNK inhibitor was used to further verify the role of JNK1/2 in the inhibition of cardiomyocyte hypertrophy by ANGTPL4. Our data showed that blocking JNK1/2 signaling significantly ameliorated cardiomyocyte hypertrophy and recovered the expression level of PPARα, which was impaired by the knockdown of ANGPTL4. These findings imply that JNK inhibitors may ameliorate cardiomyocyte hypertrophy by improving the expressions of FAO-related genes in PE-induced hypertrophic cardiomyocytes. Moreover, our *in vivo* study proved the opposite trend between JNK1/2 and PPARα after AB, which were consistent with those of a previous study by Vernia et al. [34] that showed that JNK1/2 signaling exhibited an inverse relationship with PPARα. Consequently, our results suggested that the JNK1/2 signaling pathway might play a pivotal role in the ANGPTL4-mediated regulation of cardiomyocyte hypertrophy.

## Conclusions

In conclusions, we discovered that down-regulation of ANGPTL4 is capable of exacerbating PE-induced cardiomyocyte hypertrophy. A potential core mechanism is that ANGPTL4 promotes the expressions of FAO-related genes via the regulation of PPARα through JNK1/2 signaling. Therefore, ANGPTL4 may be a new target for the treatment of cardiomyocyte hypertrophy; however, further research is required to uncover additional mechanisms.

## Abbreviations

ANGPTL4: angiopoietin-like protein 4
ANP: atrial natriuretic peptide
CPT-1: carnitine palmitoyl transferase-1
FAO: fatty acid oxidation
JNK1/2: c-Jun NH2-terminal Kinase 1/2
LPL: lipoprotein lipase
PPARα: peroxisome proliferator-activated receptor α

## References

[1] Sheng H., Zhu J., Wu X. and Zhang J. (2008) Blockade of calcineurin reverses cardiac hypertrophy and induces the down-regulation of JNK mRNA expression in renovascular hypertensive rats. J Renin Angiotensin Aldosterone Syst. 9, 139–145 10.1177/1470320308096048 18957384

[2] Opie L.H. and Knuuti J. (2009) The adrenergic-fatty acid load in heart failure. J. Am. Coll. Cardiol. 54, 1637–1646 10.1016/j.jacc.2009.07.024 19850204

[3] Kolwicz S.C., Purohit S. and Tian R. (2013) Cardiac metabolism and its interactions with contraction, growth, and survival of cardiomyocytes. Circ. Res. 113, 603–616 10.1161/CIRCRESAHA.113.302095 23948585 PMC3845521

[4] Aubert G., Martin O.J., Horton J.L., Lai L., Vega R.B., Leone T.C. (2016) The failing heart relies on ketone bodies as a fuel. Circulation 133, 698–705 26819376 10.1161/CIRCULATIONAHA.115.017355 PMC4766035

[5] Chokshi A., Drosatos K., Cheema F.H., Ji R., Khawaja T., Yu S. (2012) Ventricular assist device implantation corrects myocardial lipotoxicity, reverses insulin resistance, and normalizes cardiac metabolism in patients with advanced heart failure. Circulation 125, 2844–2853 10.1161/CIRCULATIONAHA.111.060889 22586279 PMC3464497

[6] Bedi K.C. Jr, Snyder N.W., Brandimarto J., Aziz M., Mesaros C., Worth A.J. (2016) Evidence for intramyocardial disruption of lipid metabolism and increased myocardial ketone utilization in advanced human heart failure. Circulation 133, 706–716 10.1161/CIRCULATIONAHA.115.017355 10.1161/CIRCULATIONAHA.115.017355 26819374 PMC4779339

[7] Lei X., Shi F., Basu D., Huq A., Routhier S., Day R. (2011) Proteolytic processing of angiopoietin-like protein 4 by proprotein convertases modulates its inhibitory effects on lipoprotein lipase activity. J. Biol. Chem. 286, 15747–15756 10.1074/jbc.M110.217638 21398697 PMC3091183

[8] Lee E.C., Desai U., Gololobov G., Hong S., Feng X., Yu X.C. (2009) Identification of a new functional domain in angiopoietin-like 3 (ANGPTL3) and angiopoietin- like 4 (ANGPTL4) involved in binding and inhibition of lipoprotein lipase (LPL). J. Biol. Chem. 284, 13735–13745 10.1074/jbc.M807899200 19318355 PMC2679475

[9] Li Y., Chen C., Yao F., Su Q., Liu D., Xue R. (2014) AMPK inhibits cardiac hypertrophy by promoting autophagy via mTORC1. Arch. Biochem. Biophys. 558, 79–86 10.1016/j.abb.2014.06.023 25009141

[10] Dong B., Xue R., Sun Y., Dong Y. and Liu C. (2017) Sestrin 2 attenuates neonatal rat cardiomyocyte hypertrophy induced by phenylephrine via inhibiting ERK1/2. Mol. Cell. Biochem. 433, 113-123. 433, 113–123 10.1007/s11010-017-3020-2 28497371

[11] Xue R., Zeng J., Chen Y., Chen C., Tan W., Zhao J. (2017) Sestrin 1 ameliorates cardiac hypertrophy via autophagy activation. J. Cell. Mol. Med. 21, 1193–1205 10.1111/jcmm.13052 28181410 PMC5431155

[12] Liu C., Xue R., Wu D., Wu L., Chen C., Tan W. (2014) REDD1 attenuates cardiac hypertrophy via enhancing autophagy. Biochem. Biophys. Res. Commun. 454, 215–220 10.1016/j.bbrc.2014.10.079 25450383

[13] Hajri T. and Abumrad NA. (2002) Fatty acid transport across membranes: relevance to nutrition and metabolic pathology. Annu. Rev. Nutr. 22, 383–415 10.1146/annurev.nutr.22.020402.130846 12055351

[14] Luiken J.J., Coort S.L., Koonen D.P., van der Horst D.J., Bonen A., Zorzano A. (2004) Regulation of cardiac long-chain fatty acid and glucose uptake by translocation of substrate transporters. Pflugers Arch. 448, 1–15 14872244 10.1007/s00424-003-1199-4

[15] Burns K.A. and Vanden Heuvel JP. (2007) Modulation of PPAR activity via phosphorylation. Biochim. Biophys. Acta 1771, 952–960 10.1007/s00424-003-1199-4 10.1007/s00424-003-1199-4 17560826 PMC2712836

[16] Meng R., Pei Z., Zhang A., Zhou Y., Cai X., Chen B. (2011) AMPK activation enhances PPARα activity to inhibit cardiac hypertrophy via ERK1/2 MAPK signaling pathway. Arch. Biochem. Biophys. 511, 1–7 10.1016/j.abb.2011.04.010 21530483

[17] Stapleton C.M., Joo J.H., Kim Y.S., Liao G., Panettieri R.A. and Jetten A.M. (2010) Induction of ANGPTL4 expression in human airway smooth muscle cells by PMA through activation of PKC and MAPK pathways. Exp. Cell. Res. 316, 507–516 10.1016/j.yexcr.2009.12.004 20025870 PMC2815125

[18] Choi S.S., Park J. and Choi J.H. (2014) Revisiting PPARγ as a target for the treatment of metabolic disorders. BMB Rep. 47, 599–608 10.5483/BMBRep.2014.47.11.174 25154720 PMC4281338

[19] Finck B.N., Lehman J.J., Leone T.C., Welch M.J., Bennett M.J., Kovacs A. (2002) The cardiac phenotype induced by PPARalpha overexpression mimics that caused by diabetes mellitus. J. Clin. Invest. 109, 121–130 10.1172/JCI0214080 11781357 PMC150824

[20] Sukonina V., Lookene A., Olivecrona T. and Olivecrona G. (2006) Angiopoietin-like protein 4 converts lipoprotein lipase to inactive monomers and modulates lipase activity in adipose tissue. Proc. Natl. Acad. Sci. U.S.A. 103, 17450–17455 10.1073/pnas.0604026103 17088546 PMC1859949

[21] Koliwad S.K., Kuo T., Shipp L.E., Gray N.E., Backhed F., So A.Y. (2009) Angiopoietin-like 4 (ANGPTL4, fasting-induced adipose factor) is a direct glucocorticoid receptor target and participates in glucocorticoid-regulated triglyceride metabolism. J. Biol. Chem. 284, 25593–25601 10.1074/jbc.M109.025452 19628874 PMC2757961

[22] Shan L., Yu X.C., Liu Z., Hu Y., Sturgis L.T., Miranda M.L. (2009) The angiopoietin-like proteins ANGPTL3 and ANGPTL4 inhibit lipoprotein lipase activity through distinct mechanisms. J. Biol. Chem. 284, 1419–1424 10.1074/jbc.M808477200 19028676 PMC3769808

[23] Barger P.M. and Kelly DP. (2000) PPAR signaling in the control of cardiac energy metabolism. Trends Cardiovasc. Med. 10, 238–245 10.1016/S1050-1738(00)00077-3 11282301

[24] Wang M., Wang J., Tan R., Wu Q., Qiu H., Yang J. (2013) Effect of berberine on PPARα/NO activation in high glucose-and insulin-induced cardiomyocyte hypertrophy. Evid. Based Complement. Alternat. Med. 2013, 285489 23573121 10.1155/2013/285489 PMC3616349

[25] Liang F., Wang F., Zhang S. and Gardner D.G. (2003) Peroxisome proliferator activated receptor (PPAR)alpha agonists inhibit hypertrophy of neonatal rat cardiac myocytes. Endocrinology 144, 4187–4194 10.1210/en.2002-0217 12933694

[26] Lehman J.J. and Kelly DP. (2002) Gene regulatory mechanisms governing energy metabolism during cardiac hypertrophic growth. Heart Fail. Rev. 7, 175–185 10.1023/A:1015332726303 11988641

[27] Boudina S. and Abel ED. (2010) Diabetic cardiomyopathy, causes and effects. Rev. Endocr. Metab. Disord. 11, 31–39 10.1007/s11154-010-9131-7 20180026 PMC2914514

[28] Smeets P.J., Teunissen B.E., Willemsen P.H., van Nieuwenhoven F.A., Brouns A.E., Janssen B.J. (2008) Cardiac hypertrophy is enhanced in PPAR alpha-/- mice in response to chronic pressure overload. Cardiovasc. Res. 78, 79–89 18187461 10.1093/cvr/cvn001

[29] Goikoetxea M.J., Beaumont J., González A., López B., Querejeta R., Larman M. (2006) Altered cardiac expression of peroxisome proliferator-activated receptor-isoforms in patients with hypertensive heart disease. Cardiovasc. Res. 69, 899–907 10.1093/cvr/cvn001 10.1093/cvr/cvn001 16371224

[30] Kersten S., Mandard S., Tan N.S., Escher P., Metzger D., Chambon P. (2000) Characterization of the fasting-induced adipose factor FIAF, a novel peroxisome proliferator-activated receptor target gene. J. Biol. Chem. 275, 28488–28493 10.1074/jbc.M004029200 10862772

[31] Mandard S. I, Zandbergen F., Tan N.S., Escher P., Patsouris D., Koenig W. (2004) The direct peroxisome proliferator-activated receptor target fasting-induced adipose factor (FIAF/PGAR/ANGPTL4) is present in blood plasma as a truncated protein that is increased by fenofibrate treatment. J. Biol. Chem. 279, 34411–34420 10.1074/jbc.M403058200 15190076

[32] Romeo S., Yin W., Kozlitina J., Pennacchio L.A., Boerwinkle E., Hobbs H.H. (2009) Rare loss-offunction mutations in ANGPTL family members contribute to plasma triglyceride levels in humans. J. Clin. Invest. 119, 70–79 19075393 10.1172/JCI37118 PMC2613476

[33] Lopaschuk G.D., Ussher J.R., Folmes C.D., Jaswal J.S. and Stanley W.C. (2010) Myocardial fatty acid metabolism in health and disease. Physiol. Rev. 90, 207–258 10.1152/physrev.00015.2009 20086077

[34] Vernia S., Cavanagh-Kyros J., Garcia-Haro L., Sabio G., Barrett T., Jung D.Y. (2014) The PPARα-FGF21 hormone axis contributes to metabolic regulation by the hepatic JNK signaling pathway. Cell Metab. 20, 512–525 10.1016/j.cmet.2014.06.010 25043817 PMC4156535

